# Clinical and Radiological Outcomes at ≥10-Year Follow-up After Matrix-induced Autologous Chondrocyte Implantation in the Patellofemoral Joint

**DOI:** 10.1177/03635465241262337

**Published:** 2024-08-05

**Authors:** Jay R. Ebert, Sven Klinken, Michael Fallon, David J. Wood, Gregory C. Janes

**Affiliations:** † School of Human Sciences (Exercise and Sport Science), University of Western Australia, Crawley, Western Australia, Australia; ‡ HFRC Rehabilitation Clinic, Nedlands, Western Australia, Australia; § Perth Radiological Clinic, Subiaco, Western Australia, Australia; ‖ Medical School, University of Western Australia, Crawley, Western Australia, Australia; ¶ Perth Orthopaedic & Sports Medicine Centre, West Perth, Western Australia, Australia; Investigation performed at the University of Western Australia, Crawley, Western Australia, Australia

**Keywords:** matrix-induced autologous chondrocyte implantation (MACI), patellofemoral joint, clinical outcomes, magnetic resonance imaging (MRI)

## Abstract

**Background:**

Matrix-induced autologous chondrocyte implantation (MACI) has demonstrated encouraging outcomes in the treatment of knee cartilage defects, although limited research is available on its longer term (≥10 years) sustainability in the patellofemoral joint.

**Purpose:**

To report the clinical and radiological outcomes at ≥10 years in a prospectively recruited cohort of patients undergoing MACI in the patellofemoral joint and compare outcomes in patients undergoing MACI on the patella versus the trochlea.

**Study Design:**

Case series; Level of evidence, 4.

**Methods:**

The current study prospectively enrolled 95 patients who underwent patellofemoral MACI, of whom 29 (13 patella, 16 trochlea) underwent concomitant tibial tubercle osteotomy. Patients were assessed preoperatively and at 2, 5, and ≥10 years using a range of patient-reported outcome measures (PROMs) including the Knee injury and Osteoarthritis Outcome Score, the 36-item Short Form Health Survey, and the frequency and severity of knee pain as well as patient satisfaction, full active knee flexion and extension, and peak isokinetic knee extensor and flexor torques. High-resolution magnetic resonance imaging (MRI) was performed to assess pertinent graft parameters, as well as determine an overall MRI composite score, per the Magnetic Resonance Observation of Cartilage Repair Tissue scoring system. Results were analyzed according to the graft location (patella or trochlea).

**Results:**

Of the 95 patients recruited, 82 patients (41 patella, 41 trochlea) were available for a clinical review at ≥10 years after surgery (mean follow-up, 11.9 years [range, 10-15 years]). For the whole patellofemoral MACI cohort, all PROMs significantly improved over time (*P* < .05), with no significant changes (*P* > .05) observed in any MRI-based score from 2 to ≥10 years after surgery. At ≥10 years, 90.2% (n = 74) were satisfied with MACI in relieving their knee pain, and 85.4% (n = 70) were satisfied with the improvement in their ability to participate in sports. No differences (*P* > .05) were observed in PROMs between those undergoing patellar MACI and those undergoing trochlear MACI, although a significant group effect was observed for limb symmetry indices of knee extensor (*P* = .009) and flexor (*P* = .041) strength, which were greater in those undergoing patellar (vs trochlear) MACI. No statistically significant differences (*P* > .05) were observed between patellar and trochlear grafts on any MRI-based measure. In the cohort assessed at ≥10 years after surgery, 4 patients (2 patella, 2 trochlea) demonstrated graft failure on MRI scans, although a further 3 patients (all trochlea) were omitted from the ≥10-year review for having already progressed to total knee arthroplasty.

**Conclusion:**

Good clinical scores, high levels of patient satisfaction, and adequate graft survivorship were observed at ≥10 years after MACI on the patella and trochlea.

Matrix-induced autologous chondrocyte implantation (MACI) has demonstrated encouraging midterm clinical and magnetic resonance imaging (MRI) outcomes,^4,5,11,12,16,34^ with limited studies also demonstrating good outcomes at 10 years and beyond.^1,7,10,17,28^ Of interest, these longer term (>10 years) studies have generally reported outcomes in smaller patient cohorts, have evaluated outcomes across mixed repair sites, and/or have lacked postoperative MRI,^1,7,17,28^ and none has focused on the patellofemoral knee joint.

A systematic review reported that numerous patellofemoral cartilage restoration techniques resulted in functional improvement and low failure rates, although conclusions could not be made as to which technique was best.^2^ However, a recently published treatment algorithm for knee cartilage defects highlighted the array of cartilage restoration procedures available, although the authors suggested that cell-based modalities such as MACI may be preferred in the patellofemoral joint.^21^ This is despite the relative lack of longer term data currently available on outcomes specifically in those undergoing MACI in the patellofemoral joint.

Therefore, long-term evidence is required to justify MACI, specifically when performed on the patellofemoral joint. Furthermore, given the lack of longer term data comparing outcomes after patellofemoral MACI on the patella versus the trochlea, the current study sought to investigate whether differences in clinical outcomes or graft survivorship exist to provide the clinician with evidence to enhance patient education and offer realistic expectations to the patient on longer term outcomes that are more specific to the operative location. The aims of the current study were to conduct a prospective clinical and MRI-based evaluation at ≥10 years after surgery in a large cohort of patients who underwent patellofemoral MACI and investigate any postoperative differences that may exist in clinical outcomes and graft status in those who underwent MACI on the patella or trochlea. It was hypothesized that (1) patients would significantly improve in clinical outcomes from preoperatively to ≥10 years after patellofemoral MACI, (2) measures of graft status would not demonstrate a significant deterioration from 2 to ≥10 years after surgery as assessed using MRI, and (3) no significant clinical or MRI-based differences would be observed between MACI on the patella and MACI on the trochlea.

## Methods

### Participants

A retrospective review of prospectively recruited patients who underwent MACI on the patellofemoral joint was conducted. Patient recruitment and subsequent surgery were undertaken between November 2003 and February 2012 after receiving approval from the Hollywood Private Hospital (HPH145) Human Research Ethics Committee and after obtaining written informed consent from each patient.

Patients were deemed candidates for patellofemoral MACI and subsequently recruited if they had full-thickness, symptomatic, and isolated grade 3 or 4 patellofemoral chondral lesions, as assessed using the International Cartilage Repair Society classification system.^6^ Although surgical (and study) inclusion was permitted for patients presenting with both patellar and trochlear lesions (providing they were not direct “kissing lesions”), no patient in the cohort demonstrated simultaneous lesions on both the patella and trochlea that warranted a surgical intervention for both. Patients also had to be between 15 and 65 years of age and were considered able and willing to adhere to the rehabilitation program. Furthermore, a period of 3 to 6 months of nonoperative management (including exercise rehabilitation and lower limb strengthening) was generally advocated before considering MACI, with patients recruited by the orthopaedic surgeon (including D.J.W. and G.C.J.). These patients must have already undergone MRI and had a minimum duration of symptoms of ≥12 months.

Patients were excluded if they had ligamentous instability, had varus/valgus abnormalities (>3° tibiofemoral anatomic angle), had undergone previous extensive meniscectomy, or had ongoing progressive inflammatory arthritis. Therefore, all patients underwent preoperative MRI to evaluate the chondral defect as well as any other intra-articular abnormality. Even though patellofemoral alignment was assessed clinically by the orthopaedic specialist to evaluate patellar tracking, subluxations, lateral retinacular tightness, and the Q-angle, all patients also underwent computed tomography to assess for patellofemoral knee joint malalignment. The tibial tubercle–trochlear groove distance was measured on computed tomography, and tibial tubercle osteotomy was performed in patients with >0.9-cm lateralization of the tibial tuberosity.

### Surgical Technique and Rehabilitation

The surgical technique has been previously described.^9^ Briefly, patients initially underwent arthroscopic surgery to harvest a sample of articular cartilage from a nonweightbearing area of the knee, which was sent to the laboratory (Genzyme) for chondrocyte isolation, culturing, and subsequent seeding onto a type I/III collagen membrane (ACI-Maix; Matricel) before reimplantation. At the time of reimplantation via open arthrotomy, the chondral defect was prepared and measured (to appropriately size the membrane), with the membrane fixed with fibrin glue to the subchondral bone. In patients requiring patellofemoral realignment, combined patellofemoral lateral retinacular release and anteromedial tibial tubercle osteotomy was performed concurrently with MACI using the Heatley modification^20^ of the Fulkerson technique.^14,15^ The tibial tubercle was medialized and secured using two 3.5-mm cortical screws, positioning the patella centrally in the trochlear groove at 20° of knee flexion.

The rehabilitation protocol undertaken has also been previously described.^9^ Early-stage postoperative management was focused on passive- and active-assisted knee range of motion and restricted weightbearing within predetermined limits, activities to encourage lower extremity circulation, isometric muscular contractions to minimize muscle loss, joint icing and limb elevation for pain and swelling control, and knee bracing over a 6- to 12-week period as required. Weightbearing capacity was progressively increased, with full weightbearing permitted from 6 weeks after surgery. All patients participated in a structured outpatient rehabilitation program that was focused on improving knee range of motion, lower limb strength, and functional capacity^9^ and was individually modified as required based on defect location/size, concomitant surgery, and/or patient progression.

### Clinical Assessment

For the current study, patient-reported outcome measures (PROMs) were administered preoperatively and at 2, 5, and ≥10 years postoperatively and included (1) the Knee injury and Osteoarthritis Outcome Score (KOOS)^30^ with the Pain, Symptoms, Activities of Daily Living, Sport and Recreation, and Quality of Life subscales; (2) the 36-item Short Form Health Survey (SF-36) with a mental component summary and a physical component summary^3^; and (3) a visual analog scale (VAS) to assess the frequency and severity of pain from 0 to 10. A patient satisfaction questionnaire was employed at the ≥10-year review to assess patients’ level of satisfaction with the MACI procedure overall as well as their satisfaction with MACI in relieving knee pain, improving their ability to perform normal daily activities, and improving their ability to participate in sport. Furthermore, at 2, 5, and ≥10 years postoperatively, full active knee flexion and extension (in degrees) of the operated knee were assessed, while peak isokinetic quadriceps and hamstring torques (in N·m) were assessed on both the operated and nonoperated limbs using an isokinetic dynamometer (Isosport) at an angular velocity of 90 deg/s. All objective measurements were undertaken (and PROM scores collected) at the same private outpatient therapy clinic by a qualified therapist (J.R.E.) with extensive experience in both clinic and research settings.

### MRI Assessment

High-resolution MRI was undertaken at 2, 5, and ≥10 years postoperatively using a 1.5- or 3.0-T scanner (Symphony; Siemens) to assess grafts per the Magnetic Resonance Observation of Cartilage Repair Tissue (MOCART) scoring system.^23,29,31,33^ Standardized proton density–weighted and T2-weighted fat-saturated images were obtained in the coronal and sagittal planes (slice thickness: 3 mm; field of view: 14-15 cm; 512 matrix in at least 1 axis for proton density–weighted images with a minimum 256 matrix in 1 axis for T2-weighted images). Axial proton density–weighted fat-saturated images were also obtained (slice thickness: 3-4 mm; field of view: 14-15 cm; minimum 224 matrix in at least 1 axis). The MOCART scoring tool was used to assess pertinent parameters of graft repair (graft infill, signal intensity, border integration, surface contour, tissue structure, effusion, subchondral lamina, and subchondral bone),^24^ with each scored individually from 1 to 4 (1 = poor, 2 = fair, 3 = good, 4 = excellent) in comparison to adjacent native cartilage. An overall MRI composite score was also calculated by multiplying each score by a weighting factor^29^ and then summing the scores.^8^ The MRI evaluation was performed by an independent experienced musculoskeletal radiologist (S.K.) with extensive experience in cartilage repair studies and in using the MOCART scoring tool.

### Data and Statistical Analyses

Initially, means and ranges were calculated and presented for the entire cohort undergoing patellofemoral MACI for patient (age, weight, body mass index, sex distribution), injury (duration of symptoms), and surgical (defect size, previous procedures, requirement for tibial tubercle osteotomy) variables. Furthermore, these variables were statistically compared between patients undergoing patellar MACI and those undergoing trochlear MACI via independent *t* test or chi-square test for categorical data.

For all PROMs, objective outcomes, and MRI-based outcomes, means, standard deviations, and ranges at the assessment time points (before surgery and 2, 5, and ≥10 years) were presented for the whole patellofemoral MACI cohort as well as those specifically undergoing patellar and trochlear MACI. For isokinetic strength measurements, the limb symmetry index (LSI; a measure of the operated limb as a percentage of the nonoperated limb) was calculated. Repeated-measures analysis of variance was employed to assess the change in the whole patellofemoral MACI cohort over time as well as differences between the patellar and trochlear MACI groups over time on all measures. Where a significant group or interaction effect was found, post hoc independent *t* test was used to determine time points at which the 2 groups differed.

Graft failures were reported, which in the current study were defined as graft delamination or no discernible graft tissue on MRI scans (ie, a score of 1 [poor] indicating the subchondral bone was exposed per the variable “graft infill” in the MOCART score). Statistical analysis was performed using SPSS software (Version 27.0; IBM), with significance determined at *P* < .05.

## Results

**Figure 1.**
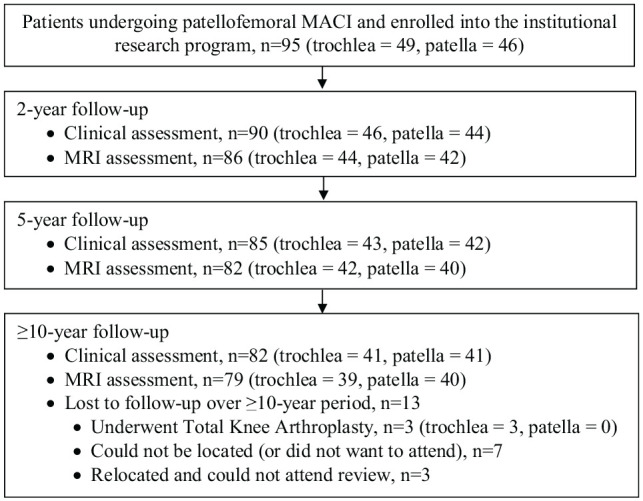
Flowchart demonstrating patients undergoing patellofemoral matrix-induced autologous chondrocyte implantation (MACI) who were enrolled in the study and were subsequently evaluated over the ≥10-year postoperative period. MRI, magnetic resonance imaging.

Throughout the recruitment period, 95 patients were enrolled and subsequently underwent MACI on the patellofemoral knee joint (Figure 1). Concomitant tibial tubercle osteotomy was undertaken in 29 patients (13 patella, 16 trochlea) (Table 1). Other concomitant surgical procedures documented within the cohort included lateral release (n = 21) and partial meniscectomy (n = 5). Although subsequent reviews of the cohort were undertaken at 2 years (n = 90) and 5 years (n = 85) after surgery, the current study included 82 patients (41 patella, 41 trochlea) who were assessed clinically at a minimum of 10 years (mean, 11.9 years [range, 10-15 years]) after surgery (Figure 1). Those who were not reviewed at ≥10 years had undergone total knee arthroplasty (TKA; n = 3), had relocated and could not attend the review (n = 3), or could not be located or did not want to attend (n = 7) (Figure 1). The patient and surgical information of the cohort assessed at ≥10 years is provided in Table 1.

**Table 1:** Patient, Injury, and Surgical Characteristics^*a*^

	All (n = 82)	Trochlea (n = 41)	Patella (n = 41)	*P* Value
Final follow-up, y	11.9 (10.0-15.0)	11.8 (10.0-15.0)	12.0 (10.5-15.0)	.781
Age at surgery, y	36.3 (20-61)	36.3 (20-54)	36.2 (23-61)	.944
Weight, kg	80.0 (46-117)	82.0 (46-102)	77.7 (47-117)	.160
Body mass index	25.5 (18.4-33.8)	25.7 (18.4-32.0)	25.1 (19.6-33.8)	.447
Defect size, cm^2^	3.4 (1.0-7.2)	3.5 (1.0-7.2)	3.2 (1.5-6.0)	.394
No. of previous procedures	1.0 (0-4)	1.0 (0-3)	1.0 (0-4)	.995
Concomitant tibial tubercle osteotomy	29 (35.4)	16 (39.0)	13 (31.7)	.488
Duration of symptoms, y	7.4 (1-25)	6.6 (1-22)	8.3 (1-25)	.186
Male sex	53 (64.6)	30 (73.2)	23 (56.1)	.013

a Data are shown as mean (range) or n (%).

### Clinical and MRI-based Outcomes

#### Whole Patellofemoral MACI Cohort

For the whole patellofemoral MACI cohort, all KOOS subscores significantly improved (*P* < .0001) from preoperatively to postoperatively, as did the physical component summary (*P* < .0001) and mental component summary (*P* = .013) scores of the SF-36 and the VAS-frequency (*P* < .0001) and VAS-severity (*P* < .0001) scores. Of the 82 patients who underwent patellofemoral MACI and were assessed clinically at final follow-up, 90.2% (n = 74) were satisfied with MACI in relieving their knee pain, 85.4% (n = 70) were satisfied with the improvement in their ability to participate in sport, and 90.2% (n = 74) were satisfied with the overall result of their surgery (Table 2). From 2 years to final follow-up (≥10 years), the range of active knee extension significantly improved (*P* = .020), although no statistically significant changes (*P* > .05) were seen in the range of active knee flexion or the LSIs for peak knee extensor (quadriceps) or flexor (hamstring) strength. Finally, no significant changes (*P* > .05) were observed in any MRI-based score from 2 to ≥10 years after surgery.

**Table 2:** Patient Satisfaction^*a*^

	Very Satisfied	Somewhat Satisfied	Somewhat Dissatisfied	Very Dissatisfied	Total Satisfied
Pain relief	51	23	8	0	74 (90.2)
Improvement in ability to undertake activities of daily living	46	28	6	2	74 (90.2)
Improvement in ability to participate in recreational activities	46	25	4	7	71 (86.6)
Improvement in ability to participate in sports	28	42	3	9	70 (85.4)
Overall satisfaction	46	28	6	2	74 (90.2)

a Data are shown as No. or n (%).

#### Patellar Versus Trochlear MACI Group

**Figure 2.**
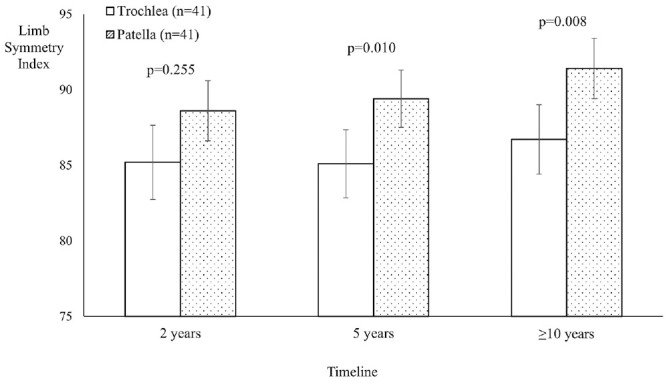
Limb symmetry indices (LSIs) for knee extensor (quadriceps) strength in the patellar and trochlear groups, with a significantly higher LSI seen in the patellar group at 5 years and final follow-up (minimum, 10 years after surgery).

**Figure 3.**
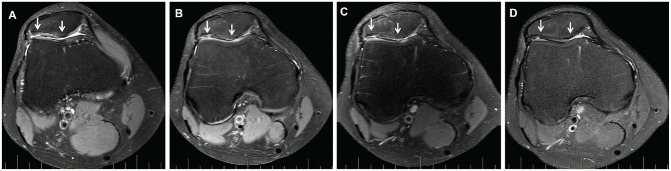
Axial proton density–weighted fat-saturated magnetic resonance imaging of a graft (between white arrows) on the patella in a patient, demonstrating (A) the preoperative chondral defect and the graft at (B) 2 years, (C) 5 years, and (D) final follow-up (minimum, 10 years after surgery).

When comparing the patellar and trochlear MACI groups, PROM scores for both groups significantly improved (*P* < .05) over the ≥10-year postoperative period, with no group differences observed (Table 3). A significant group effect was seen for the LSIs for strength, with the patellar MACI group demonstrating significantly higher LSIs for quadriceps (*P* = .009) (Table 4 and Figure 2) and hamstring (*P* = .041) (Table 4) strength compared with the trochlear MACI group. No significant differences were observed between the patellar and trochlear MACI groups on any of the MRI-based measures (Table 5). Figures 3 (patella) and 4 (trochlea) demonstrate the MRI-based progression for 2 patients enrolled in the study.

**Table 3:** Patient-reported Outcome Measure Scores^*a*^

	KOOS					SF-36		VAS	
	Pain	Symptoms	ADL	Sport	QOL	PCS	MCS	Frequency	Severity
Before surgery									
All	62.8 ± 14.8 (25.0-91.6)	65.9 ± 16.4 (17.8-100.0)	70.6 ± 16.0 (41.2-100.0)	26.7 ± 21.7 (0.0-90.0)	24.9 ± 16.4 (0.0-62.5)	35.0 ± 9.6 (19.0-58.3)	51.9 ± 8.6 (29.4-65.5)	6.5 ± 1.6 (2-9)	2.9 ± 1.4 (1-8)
Trochlea	62.6 ± 12.9 (25.0-87.5)	65.0 ± 15.0 (17.8-89.3)	70.7 ± 17.1 (44.1-97.1)	28.9 ± 20.5 (0.0-70.0)	25.4 ± 15.9 (0.0-62.5)	34.2 ± 9.9 (22.0-57.0)	51.6 ± 8.5 (29.4-63.7)	6.3 ± 1.5 (2-9)	2.8 ± 1.5 (1-8)
Patella	62.9 ± 16.7 (25.0-91.6)	66.9 ± 17.9 (35.7-100.0)	70.6 ± 15.1 (41.2-100.0)	24.5 ± 22.8 (0.0-90.0)	24.5 ± 17.1 (0.0-68.8)	35.9 ± 9.4 (19.0-58.3)	52.2 ± 8.9 (34.0-65.5)	6.6 ± 1.7 (3-9)	2.9 ± 1.3 (1-8)
2 y									
All	85.2 ± 11.8 (52.8-100.0)	87.2 ± 9.9 (57.2-100.0)	89.4 ± 10.8 (64.2-100.0)	55.0 ± 28.8 (0.0-100.0)	58.0 ± 23.3 (0.0-100.0)	47.4 ± 9.9 (24.4-60.3)	55.6 ± 6.1 (37.9-65.4)	2.0 ± 1.5 (0-7)	1.7 ± 1.1 (0-5)
Trochlea	84.2 ± 13.5 (52.8-100.0)	86.7 ± 11.2 (57.2-100.0)	88.3 ± 12.4 (64.2-100.0)	53.0 ± 31.5 (0.0-100.0)	56.7 ± 25.8 (0.0-100.0)	46.1 ± 12.2 (24.4-60.3)	55.9 ± 5.1 (41.6-61.8)	2.0 ± 1.6 (0-7)	1.7 ± 1.1 (0-4)
Patella	86.1 ± 9.9 (58.3-100.0)	87.8 ± 8.5 (67.9-100.0)	90.5 ± 8.8 (66.2-100.0)	56.9 ± 26.0 (0.0-100.0)	60.7 ± 20.5 (25.0-100.0)	48.7 ± 6.8 (29.8-57.0)	55.2 ± 7.0 (37.9-65.4)	1.9 ± 1.3 (0-5)	1.7 ± 1.2 (0-5)
5 y									
All	85.2 ± 11.8 (52.8-100.0)	87.1 ± 9.9 (57.1-100.0)	89.2 ± 10.8 (64.2-100.0)	56.3 ± 29.3 (0.0-100.0)	58.2 ± 23.5 (0.0-100.0)	47.6 ± 10.0 (24.4-60.3)	55.6 ± 6.0 (37.9-65.4)	2.0 ± 1.5 (0-7)	1.7 ± 1.2 (0-5)
Trochlea	84.2 ± 13.6 (52.8-100.0)	86.7 ± 11.2 (57.1-100.0)	88.4 ± 12.5 (64.2-100.0)	55.0 ± 31.8 (0.0-100.0)	56.3 ± 26.4 (0.0-100.0)	46.2 ± 12.3 (24.4-60.3)	56.0 ± 4.9 (41.6-61.8)	2.0 ± 1.6 (0-7)	1.8 ± 1.1 (0-4)
Patella	86.2 ± 9.8 (58.3-100.0)	87.4 ± 8.5 (67.9-100.0)	90.1 ± 9.0 (66.2-100.0)	57.5 ± 26.9 (0.0-100.0)	60.0 ± 20.4 (25.0-93.8)	48.9 ± 7.0 (29.8-57.5)	55.1 ± 6.9 (37.9-65.4)	1.9 ± 1.3 (0-5)	1.7 ± 1.2 (0-5)
≥10 y									
All	84.7 ± 12.0 (52.8-100.0)	85.9 ± 11.1 (57.1-100.0)	88.8 ± 11.1 (64.2-100.0)	57.3 ± 30.1 (0.0-100.0)	58.6 ± 23.9 (0.0-100.0)	46.7 ± 10.2 (24.4-60.3)	56.1 ± 5.6 (37.9-65.4)	2.0 ± 1.6 (0-7)	1.8 ± 1.2 (0-5)
Trochlea	83.5 ± 13.8 (52.8-100.0)	85.9 ± 12.0 (57.1-100.0)	87.6 ± 12.7 (64.2-100.0)	56.1 ± 33.1 (0.0-100.0)	57.3 ± 26.5 (0.0-100.0)	45.0 ± 12.4 (24.4-60.3)	56.5 ± 4.7 (41.6-61.8)	2.1 ± 1.8 (0-7)	1.9 ± 1.1 (0-4)
Patella	85.8 ± 10.0 (58.3-100.0)	85.9 ± 10.2 (64.3-100.0)	90.0 ± 9.4 (66.2-100.0)	58.6 ± 27.2 (0.0-100.0)	59.9 ± 21.1 (25.0-100.0)	48.3 ± 7.3 (29.8-57.0)	55.6 ± 6.4 (37.9-65.4)	1.9 ± 1.4 (0-6)	1.7 ± 1.2 (0-5)
*P* value									
Time effect	<.0001	<.0001	<.0001	<.0001	<.0001	<.0001	.013	<.0001	<.0001
Group effect	.677	.712	.619	.900	.312	.681	.510	.112	.409
Interaction effect	.815	.791	.734	.548	.533	.813	.935	.461	.518

a Data are shown as mean ± SD (range). ADL, Activities of Daily Living; KOOS, Knee injury and Osteoarthritis Outcome Score; MCS, mental component summary; PCS, physical component summary; QOL, Quality of Life; SF-36, 36-item Short Form Health Survey; Sport, Sport and Recreation; VAS, visual analog scale.

**Table 4:** Active Knee Range of Motion as Well as LSIs for Peak Knee Extensor (Quadriceps) and Flexor (Hamstring) Strength^*a*^

	Knee Extension, deg	Knee Flexion, deg	LSI for Knee Extensor Strength	LSI for Knee Flexor Strength
2 y				
All	−1.1 ± 1.5 (–5 to 2)	141.9 ± 7.0 (124 to 158)	87.1 ± 19.0 (34.7 to 112.8)	100.3 ± 14.2 (66.9 to 127.8)
Trochlea	−1.1 ± 1.2 (–3 to 1)	140.2 ± 5.8 (130 to 158)	85.2 ± 24.0 (34.7 to 112.5)	97.1 ± 12.6 (66.9 to 122.0)
Patella	−1.0 ± 1.8 (–5 to 2)	143.6 ± 7.7 (124 to 158)	88.6 ± 12.8 (64.2 to 112.8)	103.2 ± 15.3 (72.2 to 127.8)
5 y				
All	−0.9 ± 1.4 (–5 to 2)	142.1 ± 7.1 (124 to 158)	87.5 ± 18.8 (34.7 to 122.5)	100.6 ± 13.7 (66.9 to 127.8)
Trochlea	−1.0 ± 1.1 (–3 to 1)	140.6 ± 6.3 (130 to 158)	85.1 ± 23.3 (34.7 to 122.5)	97.8 ± 12.5 (66.9 to 122.0)
Patella	−0.7 ± 1.6 (–5 to 2)	143.7 ± 7.6 (124 to 158)	89.4 ± 13.5 (64.2 to 120.7)	103.1 ± 14.7 (72.2 to 127.8)
≥10 y				
All	−0.7 ± 1.5 (–5 to 5)	142.8 ± 7.8 (122 to 158)	89.0 ± 19.1 (54.7 to 138.3)	100.3 ± 14.6 (66.9 to 129.5)
Trochlea	−0.8 ± 1.5 (–5 to 5)	140.9 ± 7.3 (122 to 158)	86.7 ± 22.9 (54.7 to 138.3)	96.8 ± 12.5 (66.9 to 122.1)
Patella	−0.6 ± 1.5 (–5 to 3)	144.8 ± 7.8 (124 to 158)	91.4 ± 14.0 (64.2 to 133.3)	103.8 ± 15.8 (72.2 to 129.5)
*P* value				
Time effect	.020	.443	.393	.700
Group effect	.197	.777	.009	.041
Interaction effect	.679	.803	.531	.612

a Data are shown as mean ± SD (range). LSI, limb symmetry index.

**Table 5:** MOCART Scores^*a*^

	Graft Infill	Signal Intensity	Border Integration	Surface Contour	Tissue Structure	Subchondral Lamina	Subchondral Bone	Effusion	MRI Composite Score
2 y									
All	3.3 ± 0.8 (1-4)	3.0 ± 0.7 (1-4)	3.0 ± 1.0 (1-4)	3.2 ± 1.0 (1-4)	3.2 ± 1.0 (1-4)	3.3 ± 0.6 (2-4)	3.7 ± 0.6 (2-4)	3.8 ± 0.4 (3-4)	3.2 ± 0.6 (1.2-3.8)
Trochlea	3.3 ± 0.8 (1-4)	2.9 ± 0.6 (1-4)	3.0 ± 1.0 (1-4)	3.2 ± 1.0 (1-4)	3.1 ± 1.0 (1-4)	3.2 ± 0.6 (2-4)	3.7 ± 0.6 (2-4)	3.8 ± 0.4 (3-4)	3.2 ± 0.6 (1.2-3.8)
Patella	3.3 ± 0.8 (1-4)	3.1 ± 0.7 (1-4)	3.1 ± 1.0 (1-4)	3.3 ± 1.0 (1-4)	3.2 ± 1.0 (1-4)	3.4 ± 0.7 (2-4)	3.6 ± 0.6 (2-4)	3.7 ± 0.5 (3-4)	3.3 ± 0.6 (1.2-3.8)
5 y									
All	3.3 ± 0.8 (1-4)	3.0 ± 0.6 (1-4)	3.0 ± 1.0 (1-4)	3.1 ± 1.1 (1-4)	3.1 ± 1.0 (1-4)	3.3 ± 0.7 (2-4)	3.6 ± 0.8 (1-4)	3.7 ± 0.4 (3-4)	3.2 ± 0.6 (1.2-3.8)
Trochlea	3.3 ± 0.8 (1-4)	3.0 ± 0.6 (1-4)	3.0 ± 1.1 (1-4)	3.0 ± 1.1 (1-4)	3.1 ± 1.0 (1-4)	3.2 ± 0.6 (2-4)	3.7 ± 0.8 (1-4)	3.8 ± 0.4 (3-4)	3.1 ± 0.6 (1.2-3.8)
Patella	3.2 ± 0.8 (1-4)	3.1 ± 0.6 (1-4)	3.0 ± 1.0 (1-4)	3.2 ± 1.1 (1-4)	3.2 ± 1.0 (1-4)	3.3 ± 0.7 (2-4)	3.5 ± 0.8 (1-4)	3.7 ± 0.5 (3-4)	3.2 ± 0.6 (1.2-3.8)
≥10 y									
All	2.9 ± 0.8 (1-4)	3.0 ± 0.6 (1-4)	2.8 ± 1.1 (1-4)	2.9 ± 1.1 (1-4)	2.8 ± 1.1 (1-4)	3.2 ± 0.6 (2-4)	3.4 ± 1.0 (1-4)	3.7 ± 0.5 (3-4)	3.0 ± 0.6 (1.2-4.0)
Trochlea	2.9 ± 0.7 (1-4)	3.0 ± 0.6 (1-4)	2.8 ± 1.0 (1-4)	2.9 ± 1.1 (1-4)	2.8 ± 1.1 (1-4)	3.2 ± 0.6 (2-4)	3.6 ± 0.9 (1-4)	3.7 ± 0.5 (3-4)	3.0 ± 0.5 (1.2-3.8)
Patella	3.0 ± 0.9 (1-4)	3.1 ± 0.6 (1-4)	2.7 ± 1.1 (1-4)	2.9 ± 1.2 (1-4)	2.8 ± 1.2 (1-4)	3.3 ± 0.6 (2-4)	3.3 ± 1.1 (1-4)	3.7 ± 0.5 (3-4)	3.1 ± 0.7 (1.2-4.0)
*P* value									
Time effect	.098	.930	.232	.114	.066	.694	.147	.810	.104
Group effect	.432	.311	.812	.591	.377	.150	.102	.822	.741
Interaction effect	.801	.593	.178	.265	.600	.787	.352	.803	.522

a Data are shown as mean ± SD (range). MOCART, Magnetic Resonance Observation of Cartilage Repair Tissue; MRI, magnetic resonance imaging.

**Figure 4.**
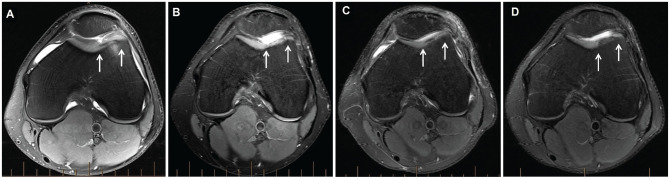
Axial proton density–weighted fat-saturated magnetic resonance imaging of a graft (between white arrows) on the trochlea in a patient, demonstrating (A) the preoperative chondral defect and the graft at (B) 2 years, (C) 5 years, and (D) final follow-up (minimum, 10 years after surgery).

### Complications, Graft Failures, and Secondary Surgical Procedures

Complications recorded at the time of (or after) surgery included wound infections (n = 2) and deep vein thrombosis (n = 2). These were treated accordingly with no further issues reported. Overall, 79 patients (40 patella, 39 trochlea) underwent MRI at final follow-up (minimum, 10 years after surgery), and 4 of these patients (2 patella, 2 trochlea) had failure as defined by graft delamination or no discernible repair tissue (MRI-based graft infill score of 1 [poor]). Furthermore, an additional 3 patients (all trochlea) were not included in the final review, as they had already progressed to TKA. Although data on nonsurgical treatment (such as intra-articular injections) that may have been undertaken after MACI were not collected, several secondary surgical procedures were recorded. These included arthroscopic debridement for subsequent meniscal abnormalities (n = 3), debridement for chondral injuries related to the MACI graft (n = 2), and debridement of the MACI graft itself (n = 1). All of these patients were retained in the analysis.

## Discussion

Limited studies have reported outcomes beyond 10 years after MACI,^1,7,10,17,28^ with current longer term evidence presenting outcomes across mixed repair sites (tibiofemoral and patellofemoral) and none focused on the patellofemoral joint. The current study demonstrated good clinical scores, high levels of patient satisfaction, and adequate graft survivorship at ≥10 years after patellofemoral MACI. Even though similar PROM and MRI-based outcomes were observed between those undergoing patellar MACI and those undergoing trochlear MACI, LSIs for knee extensor and flexor strength were greater throughout the postoperative timeline in the patellar MACI group.

A significant improvement on all PROMs was observed from before surgery to ≥10 years after surgery as expected, also reinforced by the relatively high levels of patient satisfaction reported at the final follow-up. This supports the first hypothesis, in accordance with other studies that have reported longer term clinical scores after third-generation MACI.^1,7,10,17,28^ However, these other studies have often been undertaken in smaller cohorts, lack postoperative MRI, and/or include a mixed sample of patients undergoing MACI with both tibiofemoral and patellofemoral grafts. It should be noted that around 90% of patients at ≥10 years after surgery (excluding those who had already undergone TKA) were satisfied with the overall outcome of their procedure, with 85% to 90% satisfied for each of the individual domains of pain relief, ability to undertake activities of daily living, and ability to participate in recreational and sporting activities. Therefore, around 10% remained unsatisfied, and this could be related to a number of interrelated factors including persistent pain, activity restriction, and their expectations of recovery not being met.

To the best of our knowledge, no study has reported clinical outcomes at ≥10 years after surgery in a larger prospective cohort of patients specifically undergoing MACI in the patellofemoral joint. Of interest, clinical outcomes were relatively well sustained from 2 to ≥10 years in the current study, highlighting the maintenance of clinical outcomes over a prolonged period. A natural decline in general physical function may be expected over time, which was not reflected in the PROM scores (at least on outcome measures such as the Activities of Daily Living, Sport and Recreation, and Quality of Life subscales of the KOOS as well as the physical component summary of the SF-36). Although the underlying reasons for this are not known, this may be related to the relatively young age of patients who undergo MACI or the lack of a PROM employed that is more reflective of higher level function and/or physical activity.

Previous research specifically focused on third-generation patellofemoral MACI has reported good short-term (up until 5 years) MRI-based outcomes.^9,18,19,26,32^ In support of the second hypothesis, the current study demonstrated no statistical decline in MRI-based scores from 2 to ≥10 years after surgery, and furthermore, no differences were seen between the patellar and trochlear MACI groups. Although Mestriner et al^25^ reported no differences in PROM scores or graft survivorship at a minimum of 2 years after patellofemoral MACI based on defect cause (dislocation, nontraumatic and degenerative lesion, and other), they did not directly compare patellar and trochlear MACI outcomes. Another study reported no difference in clinical or MRI-based outcomes at 2 years after patellar or trochlear MACI,^9^ although to the best of our knowledge, the current study is the first to report graft survivorship at ≥10 years after patellofemoral MACI, with comparable longer term MRI-based outcomes observed in the patella and trochlea. In addition, over the ≥10-year period, graft failure (on MRI at ≥10 years or with progression to TKA) was essentially seen in 7 patients (2 patella, 5 trochlea), thereby presenting a 5% failure rate in the patella and 12% failure rate in the trochlea.

Of interest, although there were no differences in PROMs or MRI-based outcomes (as outlined above) based on defect location (patella or trochlea), those undergoing patellar (vs trochlear) MACI had significantly higher LSIs for knee extensor and flexor strength postoperatively. Therefore, our third hypothesis was not fully supported. Knee extensor strength deficits after MACI (and patellofemoral MACI) have been previously reported^9,13,22,27^ and may be more pronounced in patients undergoing patellofemoral (vs tibiofemoral) MACI.^27^ Muller et al^27^ suggested that this may be because of the more invasive arthrotomy procedure required in undertaking patellofemoral (vs tibiofemoral) MACI, which theoretically may also be a contributing factor in those undergoing trochlear (vs patellar) MACI. However, this is unsubstantiated, and the current study lacked objective strength measurements for both the patellar and trochlear groups preoperatively. Therefore, it is unknown whether these greater postoperative deficits observed in the trochlear group were also present before surgery, despite the similar duration of symptoms.

Several limitations exist within the present study. First, it is acknowledged that the cohort in the current study was a consecutively recruited series of patients who underwent patellofemoral MACI, without any control or comparative group. Second, although a thorough history (including duration of symptoms) was collected at baseline, the cause of cartilage lesions was not documented. Even though this does not permit a comparison within the cohort (or across the patellar and trochlear groups) based on cause, it should be noted that the minimum duration of symptoms within the cohort was 12 months and was similar across the patellar and trochlear groups, and a previous study has suggested that clinical outcomes after patellofemoral autologous chondrocyte implantation are not affected by the cause of cartilage lesions.^25^ Third, the current study employed the KOOS, SF-36, and VAS, although a range of other PROMs have been employed and reported in the evaluation of patients undergoing MACI. Furthermore, activity level was not assessed in the current cohort, and given the difference between the patellar and trochlear MACI groups with respect to the postoperative LSIs for knee extensor and flexor strength, an activity-based PROM may have provided further insight into the recovery profile of the 2 groups. Finally, the difference in LSIs for strength between the patellar and trochlear MACI groups, in the absence of any other differences, should be interpreted with caution. Although a standardized rehabilitation program was undertaken in these patients, preoperative objective strength measurements were not obtained, and we do not know whether these differences in LSIs for strength may have been present before surgery, despite a similar duration of symptoms observed between the 2 groups.

## Conclusion

The current study demonstrated good clinical scores, high levels of patient satisfaction, and adequate graft survivorship, indicative of sound tissue durability at ≥10 years after patellofemoral MACI. Although LSIs for knee extensor and flexor strength were greater in those undergoing MACI to the patella, similar PROMs and MRI-based outcomes were observed between those undergoing patellar MACI and those undergoing trochlear MACI.
